# Combination of TRAIL with Bortezomib Shifted Apoptotic Signaling from DR4 to DR5 Death Receptor by Selective Internalization and Degradation of DR4

**DOI:** 10.1371/journal.pone.0109756

**Published:** 2014-10-13

**Authors:** Maxim L. Bychkov, Marine E. Gasparian, Dmitry A. Dolgikh, Mikhail P. Kirpichnikov

**Affiliations:** Department of Bioengineering, Shemyakin and Ovchinnikov Institute of Bioorganic Chemistry, Moscow, Russia; Mayo Clinic, United States of America

## Abstract

TRAIL (tumor necrosis factor-related apoptosis-inducing ligand) mediates apoptosis in cancer cells through death receptors DR4 and DR5 preferring often one receptor over another in the cells expressing both receptors. Receptor selective mutant variants of TRAIL and agonistic antibodies against DR4 and DR5 are highly promising anticancer agents. Here using DR5 specific mutant variant of TRAIL - DR5-B we have demonstrated for the first time that the sensitivity of cancer cells can be shifted from one TRAIL death receptor to another during co-treatment with anticancer drugs. First we have studied the contribution of DR4 and DR5 in HCT116 p53+/+ and HCT116 p53−/− cells and demonstrated that in HCT116 p53+/+ cells the both death receptors are involved in TRAIL-induced cell death while in HCT116 p53−/− cells prevailed DR4 signaling. The expression of death (DR4 and DR5) as well as decoy (DcR1 and DcR2) receptors was upregulated in the both cell lines either by TRAIL or by bortezomib. However, combined treatment of cells with two drugs induced strong time-dependent and p53-independent internalization and further lysosomal degradation of DR4 receptor. Interestingly DR5-B variant of TRAIL which do not bind with DR4 receptor also induced elimination of DR4 from cell surface in combination with bortezomib indicating the ligand-independent mechanism of the receptor internalization. Eliminatory internalization of DR4 resulted in activation of DR5 receptor thus DR4-dependent HCT116 p53−/− cells became highly sensitive to DR5-B in time-dependent manner. Internalization and degradation of DR4 receptor depended on activation of caspases as well as of lysosomal activity as it was completely inhibited by Z-VAD-FMK, E-64 and Baf-A1. In light of our findings, it is important to explore carefully which of the death receptors is active, when sensitizing drugs are combined with agonistic antibodies to the death receptors or receptor selective variants of TRAIL to enhance cancer treatment efficiency.

## Introduction

Tumor necrosis factor-related apoptosis-inducing ligand (TRAIL) triggers programmed cell death in various types of cancer cells without causing toxicity to normal cells [1]. Binding of TRAIL with death receptors (DR4 and DR5) induces death signals to the intracellular apoptotic machinery [2]. By contrast, two other receptors, decoy receptor DcR1 and DcR2 are unable to initiate apoptotic cell death and antagonize TRAIL-induced apoptosis [3], [4]. Many cancer cell lines express both DR4 and DR5, and each of these receptors can initiate apoptosis independently of the other. The affinity of TRAIL to the both death receptors is equal (*K*_D_ 0.5–0.9 nM) [5], [6]. However, cancer cell lines expressing DR4 and DR5 receptors at the same level often prefer one receptor to another for TRAIL signaling. The molecular basis for this selective activation via the two receptors is unknown, but the data using the receptor-specific mAbs implicated independent regulation of TRAIL signaling via homotrimeric DR4 and DR5 receptors [7], [8]. On the other hand, tumor-derived mutations in DR5 inhibited TRAIL signaling through the DR4 receptor in BJAB cells by competing for ligand binding [9].

The contribution of one death receptor versus another to apoptotic signaling is different in various tumors. Bortezomib upregulated DR5 but not DR4 receptors cell surface expression in NSCLC cell lines increasing the cell death at the same level with both DR4 and DR5 agonistic antibodies [10]. Sensitive to DR4 and DR5 agonistic antibodies chronic lymphocytic leukemia cells undergo predominantly DR4 mediated apoptosis when were pretreated with histone deacetylase inhibitors [11]. DR5-selective mutant variant of TRAIL DR5-8 exhibited greater potency in colon and breast cancer cell lines [12].

Many cancer cell lines and primary tumors are partially or fully resistant to DR4 and DR5 agonists despite the detectable expression of these receptors in cancer cells and in most tissues [13]. Anticancer drugs such as proteasome inhibitors, doxorubicin, cisplatin, HDAC inhibitors, topotecan, paclitaxel, etoposide upregulated death receptors expression in various tumor cells and sensitized cells to killing by TRAIL [14], [15]. However, these drugs usually were unable to overcome acquired resistance to TRAIL. The proteasome inhibitors can be an important exception since they allow overcoming diverse resistance mechanisms [16], [17].

Bortezomib affects TRAIL signaling at multiple levels including enhancement of TRAIL-induced caspase 8 cleavage and activation, inhibition of the cell cycle, changes in cell adherence, inhibition of NF-κB activation and overcomes TRAIL resistance in various tumor cells [18]. Induction of DR5 and/or DR4 and enhancement of TRAIL induced apoptosis by bortezomib have been demonstrated in certain types of cancer cells [19]–[21]. The maximal synergistic increase of the apoptosis by simultaneous treatment of non-small-cell lung carcinoma [21] and ovarian cancer cells [22] with bortezomib and TRAIL achieved at 16 to 24 h of exposure. Experimental results concerning the role of tumor suppressor protein p53 in stimulation of apoptosis by bortezomib are ambiguous. Bortezomib-induced apoptosis was reported to occur in a p53-independent manner in several works [23]–[25] while in a recent study [26] the sensitivity to bortezomib has been associated with p53 status.

In the present study we have demonstrated that bortezomib and TRAIL alone upregulated death and decoy receptors expression in HCT116 cells while combination of two drugs caused almost complete internalization of DR4 receptor in ligand-independent and time-dependent manner. As a result, the sensitivity of HCT116 p53^−/−^ cells shifted from DR4 to DR5 receptor. DR4 internalization was caspase dependent and lysosomal pathway of cell death was involved in this process. Our results demonstrated for the first time that the preferential receptor utilization of cancer cells during co-treatment with TRAIL and anti-neoplastic agents could be changed as regulation of receptors expression can differ from that induced by the same agents separately.

## Materials and Methods

### Cell culture and reagents

Human colorectal carcinoma cell line HCT116 p53^+/+^ and breast adenocarcinoma MDA-MB-231 cell line was from ATCC. HCT116 p53^−/−^ were kindly provided by Prof. B. Vogelstein (Johns Hopkins University School of Medicine) [27].

Cells culture medium DMEM was from PAN Biotech (Aidenbach, Germany) and fetal bovine serum (FBS) was from Hyclone (Cramlington, UK). The proteasome inhibitor bortezomib was obtained from Santa Cruz Biotechnology (Santa-Cruz, USA). General caspase inhibitor Z-VAD-FMK and inhibitor to lysosomal cathepsins E-64 were from MP Biomedicals (Eschwege, Germany), Bafilomycin A1 was from Cayman Chemicals (Ann Arbor, USA). Neutralizing antibodies to TRAIL death and decoy receptors were from Enzo Life Sciences (Farmingdale, USA) and R&D systems (Minneapolis, USA), respectively. FITC-conjugated antibodies to DR4, DR5, DcR1 and DcR2 receptor were obtained from Abnova (Walnut, USA), isotype control antibody was from Immunotech (Marcelle, France). For western blot biotinylated goat antibodies to TRAIL death and decoy receptors were purchased from R&D Systems (Minneapolis, USA), HRP streptavidin and antibodies to actin were from Sigma-Aldrich (St. Lotus, USA).

### Recombinant TRAIL preparation

Wild type and mutant variant DR5-B (containing 6 amino acid substitutions Y189N, R191K, Q193R, H264R, I266L and D269H) of TRAIL (114–281) were expressed in *E. coli* BL21(DE3) strain and purified as we previously described with some modifications [6]. Briefly, the synthetic genes of wild type and DR5-B variant were constructed into bacterial expression vector pET-32a. High-level expression of Trx/TRAIL (thioredoxin/TRAIL) fusions (approximately 150 mg from 100 ml culture) in *E. coli* strain BL-21(DE3) was induced by 0.02 mM IPTG at 28°C. Trx/TRAIL and Trx/DR5-B fusions were purified from cytoplasmic protein fraction on Ni-Sepharose high performance (GE Healthcare). After cleavage of fusions with recombinant human enteropeptidase light chain the target proteins were separated from thioredoxin on Ni-NTA agarose. To increase the yield of target proteins and reduce the amount of enteropeptidase for fusions cleavage the amino acids residue of lysine was substituted by arginine in enzyme cleavage site (Asp)_4_Lys as it was described in our previous work [28].

### MTT assay

The cytotoxic effect of wild type TRAIL, DR5 specific mutant variant DR5-B alone or in combination with bortezomib was determined by MTT assay. Briefly, cells were seeded in 96-well plates (SPL Lifesciences) at a density of 1×10^4^ per well in 200 µl culture medium (DMEM) and incubated for 16 h at 37°C. Culture medium was aspirated and 200 µl of fresh medium containing indicated concentrations of TRAIL variants and bortezomib were added to cells. Cells were incubated for indicated periods, washed with medium and MTT reagent was added at concentration 0.5 mg/ml. After incubation for 3 h, cells were centrifuged at 2000 rpm for 5 min. The supernatants were aspirated and DMSO was added to each well for formazan solubilization. Absorbance of formazan solution in wells was measured at a wavelength 540 nm using microplate reader (Bio–Rad 680) with background subtraction at 655 nm. Apoptotic cell death was confirmed by assessment of nuclear morphology after cell staining with Hoerst 33344 and propidium iodide.

### Flow cytometry

Cells were detached from culture flasks using 0.05% Trypsin-EDTA solution (PanEco), washed in ice cold PBS, and resuspended in FACS buffer (PBS with 0.1% BSA). To confirm that the trypsin does not cleave the receptors additional experiments with 0.6 mM EDTA in PBS (pH = 7.4) or Citric saline buffer (135 mM KCl, 15 mM sodium citrate, pH = 7.4) were performed (Fig. S1). TRAIL receptors cell surface expression was analyzed using FITC-conjugated mouse anti-TRAIL-R1 (DR4), anti-TRAIL-R2 (DR5), anti-TRAIL-R3 (DcR1) and anti-TRAIL-R4 (DcR2) antibodies (Abnova). Mouse IgG (Immunotech) was used as isotype control. Cells (at least 1×10^5^ cells for each sample) were incubated with 1 µg of antibodies for 1 h at 4°C, washed in ice-cold PBS twice, resuspended in FACS buffer containing 0.5 µg/ml propidium iodide and analyzed by FACScan flow cytometer using Cellquest software (Becton Dickinson).

### Western blot analysis

Cells (1×10^6^) were seeded on 100 mm cell culture dishes, trypsinized and washed by ice cold PBS twice and lysed on ice with RIPA buffer (25 mM Tris-HCl, 150 mM NaCl, 0.1% Triton X-100, 1% NP-40, 0.5% sodium deoxycholate, 0.1% SDS) supplemented with protease inhibitors. Proteins were separated by Tris-glycine SDS-PAGE (12% gel) and transferred to nitrocellulose membranes (GE Healthcare). The membrane was blocked in TBST (50 mM Tris-HCl, 150 mM NaCl with 0.01% of Tween-20) containing 5% of non-fat milk for 2 h and consequently incubated with biotinylated antibodies to TRAIL receptors and with streptavidin-HRP (Sigma) for 1 h. ECL Prime substrate (GE Healthcare) and Versa Doc MP4000 documentation system (Bio Rad) were used for visualization of TRAIL receptors. The intensity of protein bands was calculated using the ImageJ software (http://rsbweb.nih.gov/ij/, NIH, Bethesda, Maryland) by option “gel analyzer” tools.

### Analysis of TRAIL receptors internalization by confocal microscopy

To detect the localization of TRAIL receptors 5×10^4^ tumor cells were seeded overnight on glass slides in 37°C and 5% of CO_2_. Next, the culture media were replaced by a fresh one supplemented with 25 mM Baf A1 and after 1 h incubation 1 ng/mL of TRAIL variants and 1 nM of bortezomib were adjusted to the growth medium. Cells were growth for different periods in the presence of indicated reagents, washed with PBS, fixed in 3% paraformaldehyde for 30 min, permeabilized with 0.1% Triton X-100 in PBS for 10 min and blocked in 3% BSA for 30 min. FITC-conjugated antibodies to death and decoy receptors (Abnova) were added at dilution 1∶100 and cells were incubated for 1 h in the presence of Hoerst 33342 for visualization of cell nuclei. Then Non-specific bound antibodies were washed with blocking buffer containing 0.1% of Triton X-100. Cells were visualized in 0.6- µm sections using an inverted Nikon Eclipse TE2000-E laser scanning confocal microscope under a ×60 oil immersion objective.

### Statistical analysis

Data are presented as mean ± SD from at least three independent experiments. Differences between treated cells and control were assessed with the Student's *t* test, a p-value less than 0.05 was considered as statistically significant. Statistical analyses were done using Microsoft Office Excel software (Microsoft, Redmond, USA).

## Results

### Combination of bortezomib and TRAIL shifted sensitivity of HCT116 cells from DR4 to DR5 receptor in time dependent manner

**Figure 1:**
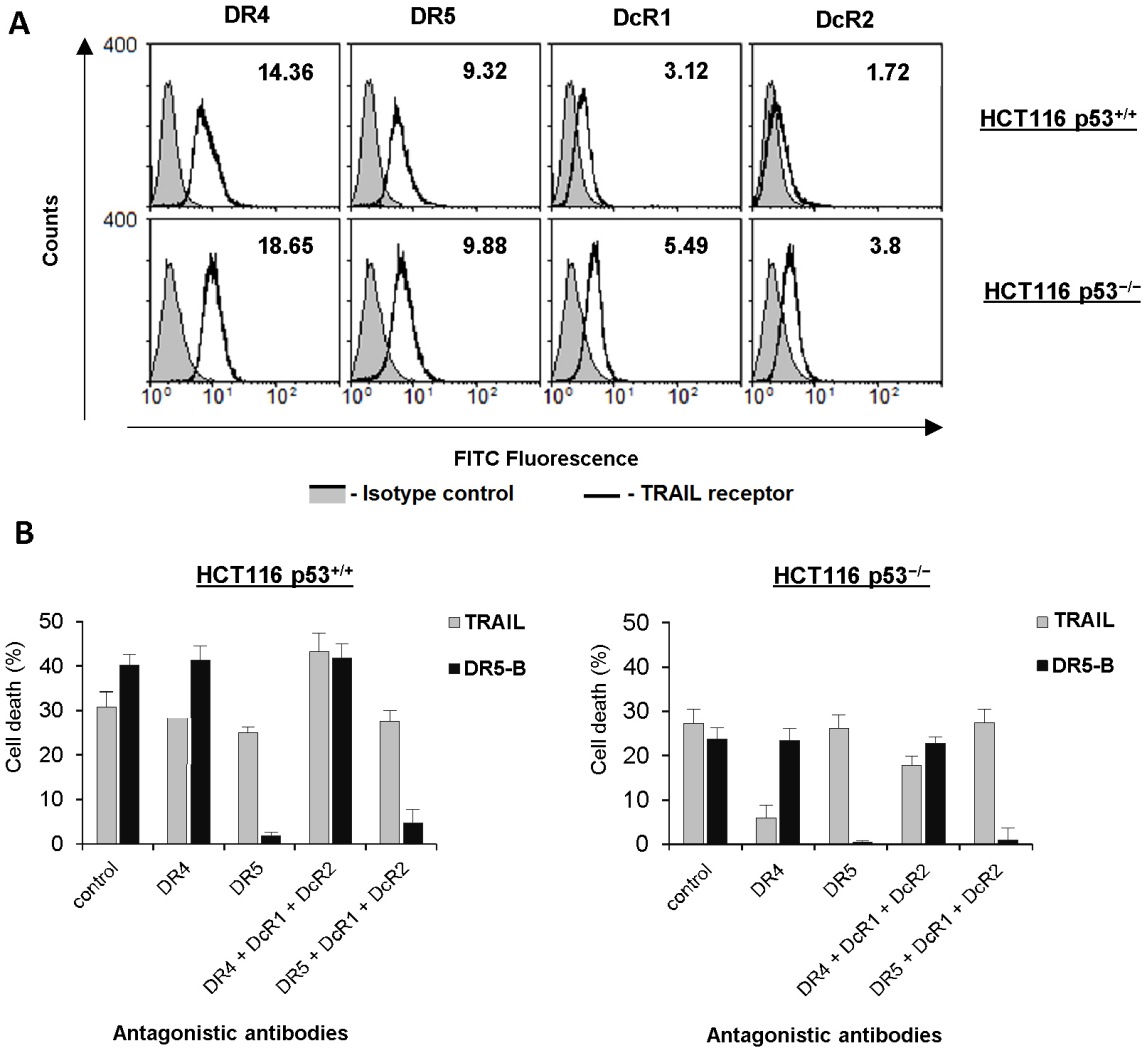
Contribution of death receptors DR4 and DR5 in TRAIL-mediated cell death in HCT116 p53^+/+^ and HCT116 p53^−/−^ cells. (A) Levels of constitutive surface expression of the death and the decoy receptors in HCT116 cells as determined by flow cytometry. (B) Cells were pre-incubated with 20 µg/ml antagonistic antibodies to death and decoy receptors or IgG1 control for 1 h following 4 h treatment with TRAIL or DR5-B (1 µg/ml) and cell death was determined by MTT test. Values are mean ± SD of at least three independent experiments.

We have chosen human colon carcinoma p53^+/+^ and p53^−/−^ HCT116 cells to investigate the contribution of death and decoy receptors in TRAIL and bortezomib induced cell death signaling. First we have analyzed the expression of the death and decoy receptors at the cells surface (Fig. 1A). TRAIL death receptors were highly expressed in these cells and the ratio of DR4 to DR5 was higher in HCT116 p53^−/−^ cells (1.7 and 2.3 for p53^+/+^ and p53^−/−^ cells, respectively). Both HCT116 p53^+/+^ and HCT116 p53^−/−^ cells expressed decoy receptors DcR1 and DcR2 at the cell surface and the level of these receptors was almost twice higher in HCT116 p53^−/−^ cells.

To investigate the contribution of DR4 and DR5 receptors to TRAIL mediated cell death we used DR5-specific mutant variant of TRAIL – DR5-B, generated in our laboratory. Earlier we have demonstrated that the dissociation constant of DR5-B to DR5 receptor was comparable to wild type TRAIL while it practically did not bind to DR4 or DcR1 receptors and its affinity to DcR2 was much lower (400 fold) in comparison to TRAIL [6]. The contribution of each receptor was analyzed using antagonistic antibodies to the death and decoy receptors (Fig. 1B). This approach revealed that TRAIL mediated cell death through both death receptors in HCT116 p53^+/+^ cells. Blocking of decoy receptors significantly increased DR5 but not DR4 signaling indicating that decoy receptors mainly inhibited the DR5 signaling in these cells. In contrast DR4 signaling was higher in HCT116 p53^−/−^ cells even when both decoy receptors were blocked by antagonistic antibodies. DR5-B variant demonstrated high specificity to DR5 receptor and was more potent in HCT116 p53^+/+^ but not in p53 null cells in comparison to TRAIL. As it was expected, blocking of decoy receptors did not affect the efficiency of the DR5-B.

**Figure 2:**
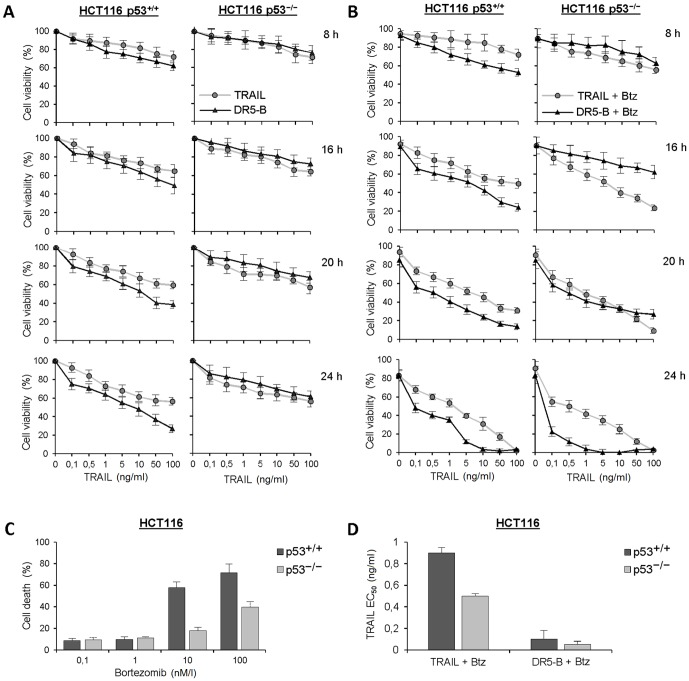
Time-depended influence of bortezomib on TRAIL or DR5-B mediated cell death in HCT116 p53^+/+^ and HCT116 p53^−/−^ cells. (A) Viability of the cells treated with different concentrations of TRAIL or DR5-B during 8, 16, 20 and 24 h of incubation. (B) Viability of the cells treated with 1 nM bortezomib and different concentrations of TRAIL or DR5-B during 8, 16, 20 and 24 h of incubation. (C) Cells were incubated with different concentrations of bortezomib and cell death was measured after 24 h of incubation. (D) Calculation of effective concentrations of TRAIL variants after 24 h treatment of cells with 1 nM bortezomib. Values are mean ± SD of at least three independent experiments.

Further, we have investigated time dependent combined action of TRAIL and bortezomib. Sensitization of TRAIL mediated cell death by bortezomib depended on ligand concentration and incubation period (Fig. 2A, 2B). In HCT116 p53^+/+^ cells the effect of bortezomib became pronounced at 8 h and reached its maximum at 24 h. In all the time points, DR5-B was more potent cell death inducer in comparison with wild type TRAIL in these cells. In HCT116 p53^−/−^ cells the behavior of DR5-B was extraordinary. Bortezomib during 16 h practically did not altered DR5-B mediated cell death but significantly increased TRAIL induced cell death. However, the efficiency of DR5-B rose strongly with time and became practically equal to TRAIL at 20 h. Moreover at 24 h of treatment DR5-B surpassed TRAIL with the half maximal effective concentration (EC_50_) 0.05±0.005 ng/ml which was almost 10 fold lower in comparison to wild type TRAIL (0.5±0.04 ng/ml) (Fig. 2D). These data indicated that combined treatment of HCT116 p53^−/−^ cells with TRAIL and bortezomib slowly (more than 16 h) shifted contribution of the receptors mediated TRAIL-induced cell death from DR4 to DR5. It should be noted that cytotoxity of bortezomib was higher in HCT116 p53^+/+^ cells in comparison to HCT116 p53^−/−^ (Fig. 2C). However its effect on TRAIL mediated cell death was higher in HCT116 p53^−/−^ cells (Fig. 2D).

### Combined treatment of HCT116 cell with bortezomib and TRAIL promotes strong DR4 receptor internalization and degradation

**Figure 3:**
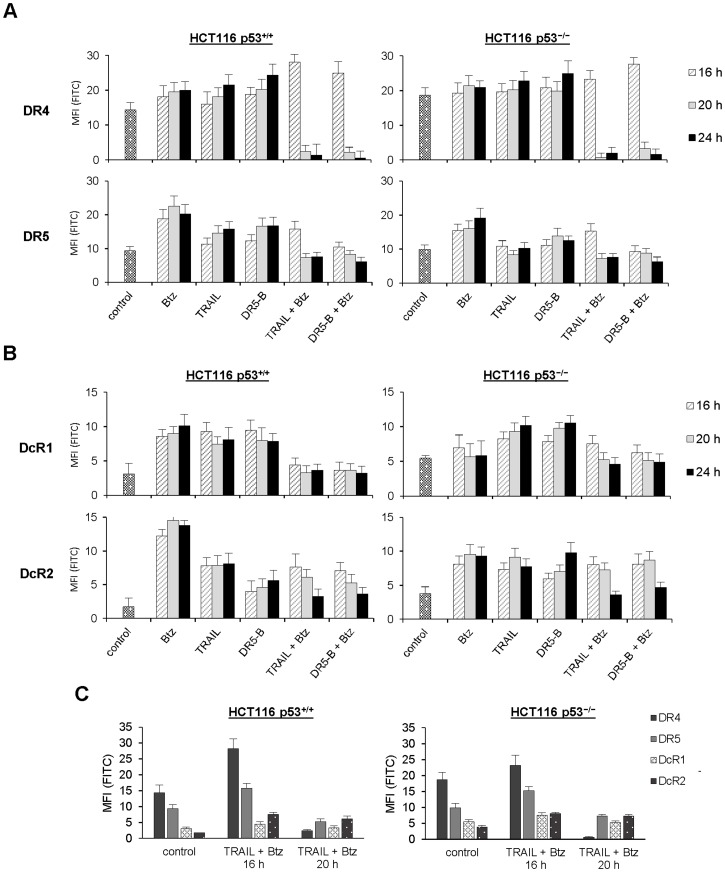
Time-dependent expression of the death and the decoy receptors at the surface of HCT116 p53^+/+^ and p53 null cells treated by bortezomib and TRAIL (or DR5-B). In all experiments, cells were treated with 1 nM bortezomib and 1 ng/ml TRAIL variants alone or in combination. (A) Profiles of the death receptors at the cell surface. (B) Profiles of the decoy receptors at the cell surface. (C) Relative level of death and decoy receptors membrane expression in cells during combined treatment with TRAIL variants and bortezomib. Values in all experiments are mean ± SD of at least three independent experiments.

To understand the nature of the shift in receptor specificity we have investigated expression of the death and the decoy cell surface receptors in HCT116 cells during treatment with bortezomib and TRAIL. Surprisingly bortezomib strongly upregulated not only the death receptors but also the decoy receptors (DcR2 more than DcR1) in HCT116 p53^+/+^ cells (Fig. 3A and B). In HCT116 p53^−/−^ cells upregulation of the receptors (with exception of DcR1) by bortezomib was less pronounced. Both TRAIL and DR5-B upregulated the decoy receptors expression in p53 independent manner (Fig. 3B). DR5 receptor was upregulated by TRAIL variants in HCT116 p53^+/+^ but not in HCT116 p53^−/−^ cells at low concentration of ligands (1 ng/ml). However at higher concentrations of ligands (more than 10 ng/ml) upregulation of DR5 receptor was detected also in HCT116 p53^−/−^ cells (data not shown).

Combined treatment with TRAIL and bortezomib induced strong increase of DR4 membrane expression during 16 h of treatment. However, during further incubation with the two drugs DR4 receptor was practically vanished from cell surface of the both HCT116 p53^+/+^ and HCT116 p53^−/−^ cells. Interestingly DR5-B, which has no affinity to DR4 receptor, induced the same effect indicating that DR4 can be internalized without ligand due to activation of DR5 signaling. These observations can explain why HCT116 p53^−/−^ cells which preferred TRAIL signaling through DR4 receptor (Fig. 1B) became DR5 sensitive after prolonged combined treatment with bortezomib and TRAIL (Fig. 2B). Finally, after 24 h of combined treatment with bortezomib, DR5-B became more effective in comparison to TRAIL in both HCT116 p53^+/+^ and HCT116 p53^−/−^ cells (Fig. 2D). This can be explained by significant increase of the relative level of the decoy and death receptors at the cell surface (Fig. 3C). Slight reduction of cell surface DR5 receptor induced by combined action of TRAIL and bortezomib could be the result of some heterotrimeric DR4/DR5 complexes internalization (Fig. 3A).

The similar phenotypic effects of specific DR4 receptor internalization during long period of combined treatment with TRAIL and bortezomib was observed in breast cancer cells MDA-MB-231where almost 5 fold reduction of DR4 receptor on the cell surface was observed after 24 h incubation (Fig. S2). In the same conditions the level of DR5 as well as decoy receptors practically were not affected. As a result, the DR4 dependent MDA-MB-231cells as it was demonstrated using antagonistic receptors to death receptors (Fig. S3A) became more sensitive to DR5-B variant in comparison to TRAIL after 16 h of incubation with bortezomib (Fig. S3B).

**Figure 4:**
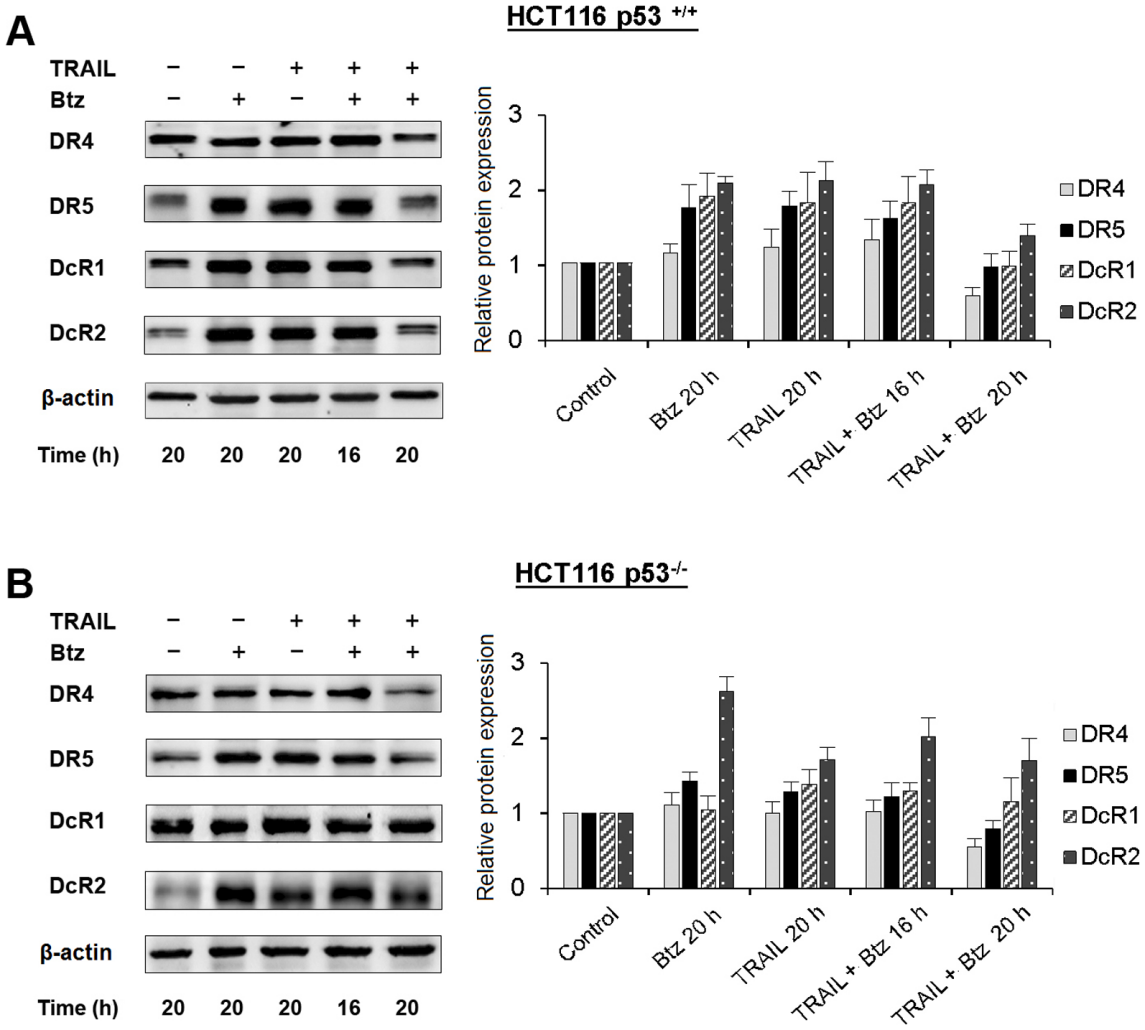
The content of death and decoy receptors in total cell extracts of HCT116 p53^+/+^ and HCT116 p53^−/−^ cells treated by TRAIL, bortezomib or by combination of both drugs. HCT116 p53^+/+^ (A) and HCT116 p53^−/−^ (B) cells were treated with TRAIL (1 ng/ml), bortezomib (1 nM), or by combination of both drugs for various time periods and the content of receptors in total cell extract was determined by Western blot analysis using appropriate biotinylated antibodies to each receptor. Densitometric analysis of three independent experiments was performed using ImageJ software. Values in all experiments are mean ± SD of at least three independent experiments.

Changes of the death and the decoy receptors expression profile in total cellular extract determined by western blot analysis practically coincided with the cell surface expression during treatment with bortezomib and TRAIL (Fig. 4A, 4B). The amount of DR4 receptor after 20 h of incubation was significantly reduced in co-treatment experiments in p53-independent manner, while the levels of DR5 and DcR1 remained almost the same as in non-treated cells. Only the level of DcR2 in total cell extract remained higher after prolonged treatment with the two drugs.

**Figure 5:**
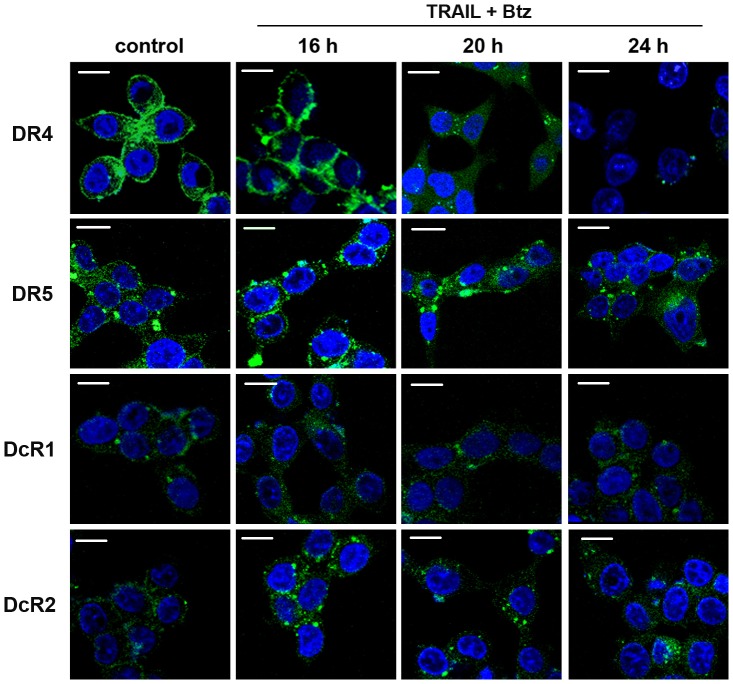
Confocal microscopic analysis of death and decoy receptors localization in HCT116 p53^−/−^ cells treated with TRAIL and bortezomib for indicated periods. Scale bar  = 10 µm. Cells were grown for different periods in the presence of indicated reagents, washed with PBS, fixed in 3% paraformaldehyde for 30 min, permeabilized with 0.1% Triton X-100 in PBS for 10 min and blocked in 3% BSA for 30 min. FITC-conjugated antibodies to DR4, DR5, DcR1 and DcR2 receptors (Abnova) were added at dilution 1∶100 and cells were incubated for 1 h in the presence of Hoerst 33342 for visualization of cell nuclei. Then non-specific bound antibodies were washed with blocking buffer containing 0.1% of Triton X-100. Cells were visualized in 0.6- µm sections using an inverted Nikon Eclipse TE2000-E laser scanning confocal microscope under a ×60 oil immersion objective.

The confocal microscopy experiments with FITC-conjugated antibodies to DR4 receptor conformed the internalization and degradation of DR4 during long treatment of cells in the presence of TRAIL and bortezomib (Fig. 5). In 20 h the major part of DR4 receptor was transported from membrane to the cytoplasm as a part of endosomes. Further incubation of the cells with two drugs (24 h of co-treatment) resulted to dramatic degradation of DR4. Partial internalization but not degradation of DR5, DcR2 and DcR1 receptors was observed during long period of incubation of the cells with bortezomib and TRAIL (Fig. 3 and 5).

**Figure 6:**
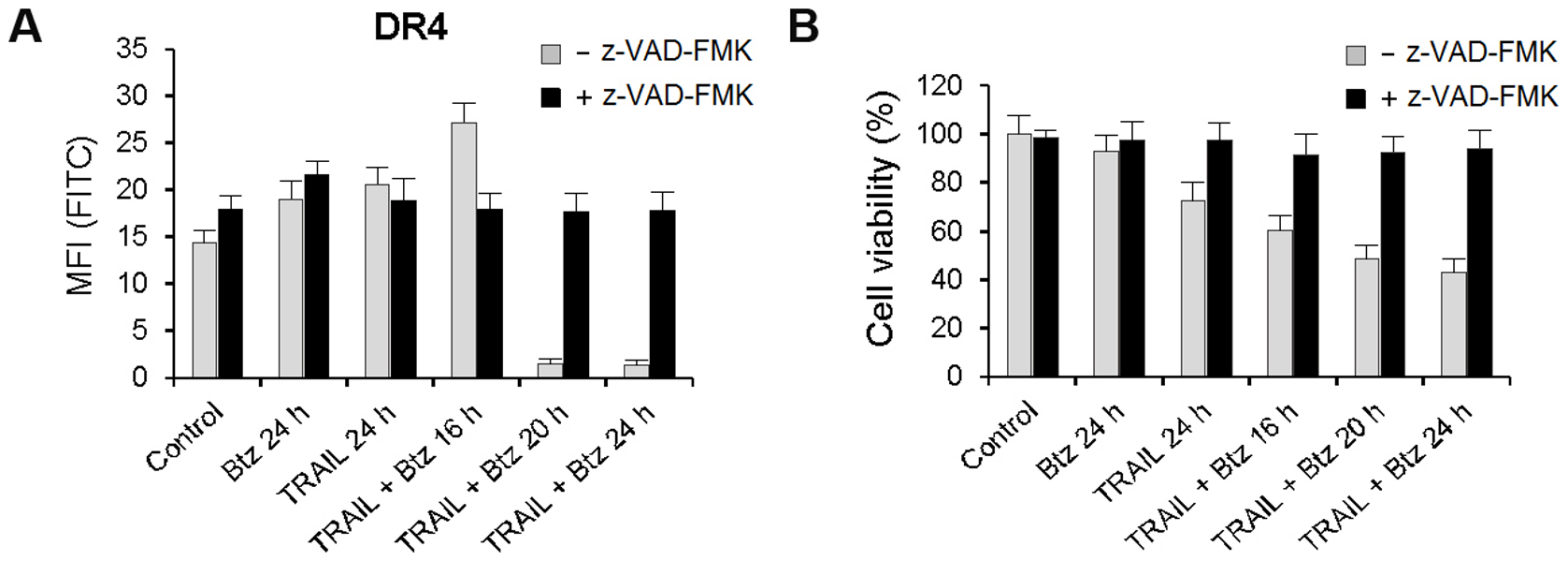
Inhibition of bortezomib and TRAIL induced DR4 internalization by z-VAD-FMK. DR4 receptor cell surface expression and viability of the HCT116 p53^+/+^ cells were analyzed after incubation with 10 µM z-VAD-FMK for 1 h following treatments with TRAIL (1 ng/ml) and bortezomib (1 nM) or in combination of the two drugs.

General caspase inhibitor z-VAD-FMK inhibited cell death and abrogated bortezomib and TRAIL induced disappearance of DR4 receptor from the cell surface indicating the essential role of caspases activation in DR4 internalization (Fig. 6). The similar effects were observed with DR5-B variant in the both HCT116 p53^+/+^ and HCT116 p53^−/−^ cells (data not shown).

### Inhibition of lysosomal activity prevented TRAIL and bortezomib induced DR4 internalization and degradation

**Figure 7:**
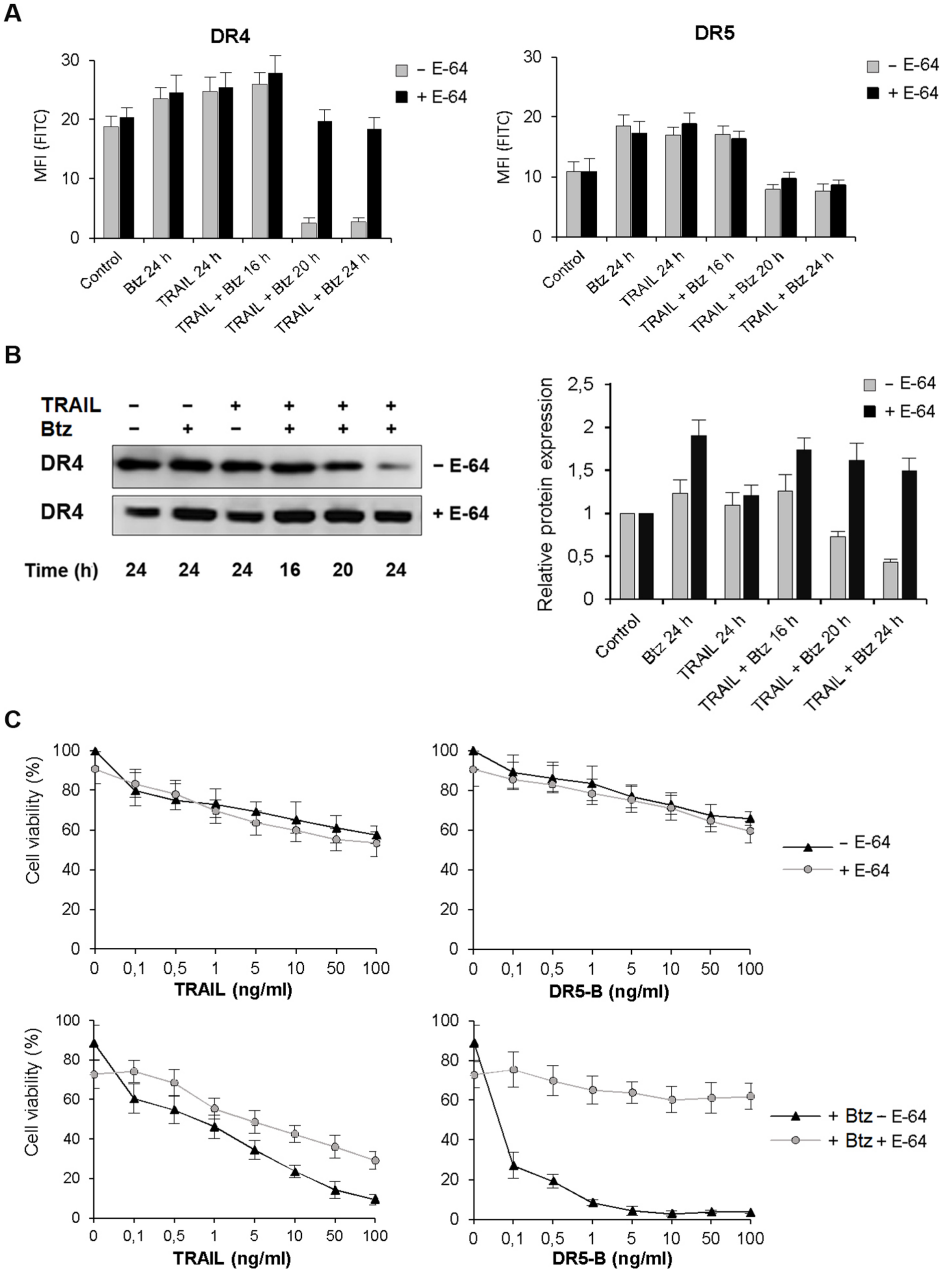
Inhibition of bortezomib and TRAIL induced DR4 internalization by E-64 in HCT116 p53^−/−^ cells. (A) DR4 and DR5 receptors cell surface expression was analyzed after incubation of the cells with E-64 (25 µM) for 1 h following medium change and treatment with TRAIL (1 ng/ml), bortezomib (1 nM) or in combination of the two drugs for 24 h. (B) Cells were treated as in (A) and the expression of DR4 and DR5 receptors in total cell extract was determined by Western blot analysis using appropriate biotinylated antibodies. Densitometric analysis of three independent experiments was performed using ImageJ software. (C) Inhibition of bortezomib and TRAIL induced cell death by E-64 in the cells was determined by MTT test. Values in all experiments are mean ± SD of at least three independent experiments.

Next, we have investigated the molecular mechanisms of bortezomib and TRAIL induced DR4 internalization and degradation by inhibition of lysosomal activity with highly selective inhibitor to lysosomal cathepsins B, H, and L E-64 and specific inhibitor of the vacuolar H(+)-ATPase bafilomycin A1 (Baf-A1) which blocks the endosome acidification. Both inhibitors prevented internalization of DR4 receptor (Fig. 7A, 8A). In the same experiments, E-64 did not affect DR5 receptor cell surface expression while Baf-A1 protected slight reduction of DR5 from cell surface induced by combined action of TRAIL and bortezomib (Fig. 3A, 8A). Probably Baf-A1 prevented not only DR4 but also some heterotrimeric DR4/DR5 complexes internalization.

**Figure 8:**
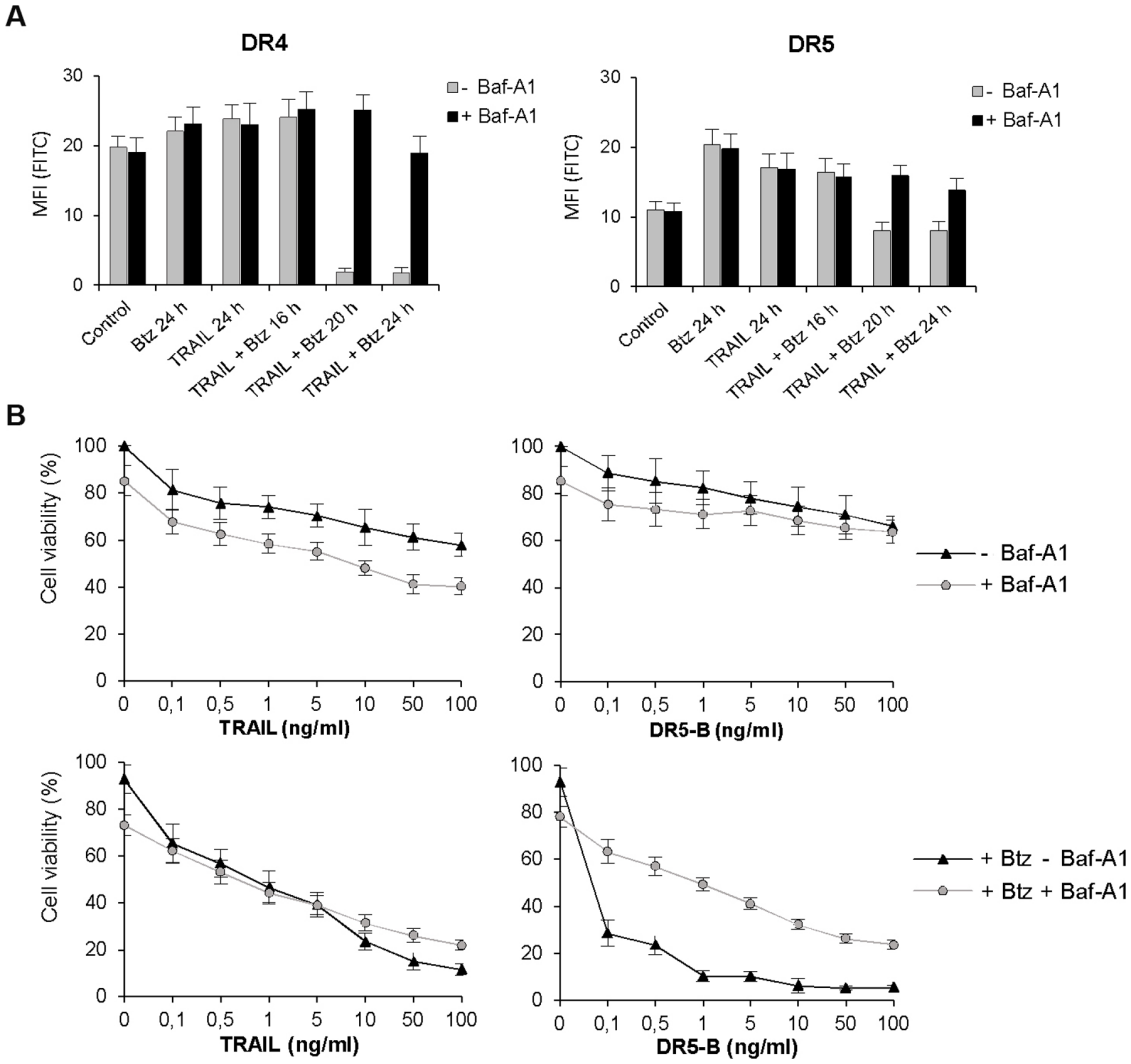
Inhibition of lysosomal activity by Baf-A1 prevented TRAIL and bortezomib induced DR4 internalization. (A) DR4 and DR5 receptors cell surface expression was analyzed in HCT116 p53^−/−^ cells pretreated with Baf-A1 (25 µM) for 1 h following bortezomib (1 nM) and TRAIL (1 ng/ml) treatment alone or in combination for 24 h. (B) Viability of the cells treated as in (A) was determined by MTT test. Values in all experiments are mean ± SD of at least three independent experiments.

Prevention of DR4 internalization by Baf-A1 and E-64 strongly inhibited the enhancing effect of bortezomib on DR5-B mediated cell death while the action of wild type TRAIL and bortezomib was only slightly decreased (Fig. 7C, 8B). E-64 itself in combination with bortezomib induced almost 30% cell death but did not significantly affect apoptosis induced by TRAIL or DR5-B (Fig. 8A, 8B). At the same time, E-64 completely inhibited the effect of bortezomib on DR5-B mediated cell death in HCT116 p53^−/−^ cells while TRAIL signaling was only partially inhibited (Fig. 7C). These are strong arguments to suggest that DR4 internalization is required for DR5 activation.

Summarizing obtained data it may be suggested that Baf-A1 and E-64 inhibited bortezomib and TRAIL/or DR5-B mediated cell death indicating that activation of lysosomal cell death pathway is involved in combined action of two agents.

## Discussion

Despite numerous studies on modulation of the death and the decoy receptors expression by cytotoxic drugs, the effects of combined treatment with TRAIL and the sensitizers were not investigated in details. In the present study, we have demonstrated for the first time that the sensitivity of cancer cells can be shifted from one TRAIL death receptor to another by chemotherapeutic drug. We have shown that DR4-dependent HCT116 p53^−/−^ and MDA-MB-231 cells became highly sensitive to DR5 specific mutant TRAIL variant DR5-B after prolonged combined treatment with bortezomib by selective internalization of DR4 receptor (Fig. 1, 2, S2A, B). We have found that combined treatment of HCT116 cells with bortezomib and TRAIL changed the death and the decoy receptors profile and these changes differed from that induced by these drugs separately. Bortezomib itself strongly upregulated not only the expression of DR5 but also DcR2 decoy receptor in HCT116 cells in p53-independent manner. DcR1 decoy receptor was also upregulated by this drug but the effect depended on p53 status (Fig. 3, 4). TRAIL alone upregulated DR5 expression at the cell surface in p53-dependent and the decoy receptors expression in p53-independent manner. Death receptor DR4 was also slightly upregulated by TRAIL or bortezomib in both HCT116 p53^+/+^ and HCT116 p53^−/−^ cells when these drugs were used separately. However, combination of these two drugs resulted in strong increase of DR4 expression at the cell surface during 16 h of treatment with following dramatic decrease in longer incubation period (more than 16 h) independently of p53 status (Fig. 3A). Importantly DR5 specific mutant variant of TRAIL DR5-B, which demonstrated no affinity to DR4 and DcR1 receptors and highly reduced affinity to DcR2 receptor [6], modulated the death and the decoy receptors expression in the same way as wild type TRAIL either alone or in combination with bortezomib. These observations indicated that modulation of the death and the decoy receptors by TRAIL can be induced without binding of the ligand to DR4 receptor. The detailed mechanism of ligand-independent selective internalization of DR4 remained unknown. Recently it was shown that DR4 is a substrate for ubiquitination by membrane-associated RING-CH-8 (MARCH-8) ligase, which had little impact on DR5 in breast carcinoma and melanoma cell lines [29]. To our knowledge selective caspase-dependent and ligand-independent depletion was never described before for any kind of receptor. The caspase-8 dependent internalization of CD95 death receptor upon binding of the ligand was observed for the first time in 2002 [30].

General caspase inhibitor Z-VAD-FMK prevented bortezomib and TRAIL induced loss of DR4 from the cell surface and inhibited cell death indicating that activation of caspases was essential for DR4 internalization (Fig. 6A). This observation allows explaining the ligand-independent mechanism of DR4 internalization, which could be caused by DR5-mediated activation of caspases. Irreversible specific inhibitor of lysosomal cathepsins E-64 and specific inhibitor of the vacuolar H(+)-ATPase Baf-A1 also suppressed DR4 surface depletion and further degradation (Fig. 7A, 8A) and strongly inhibited DR5-B and bortezomib induced cell death (Fig. 7B, 8B). These observations are indicating that bortezomib in combination with TRAIL activated lysosomes, which contributed to DR4 receptor internalization and degradation. Probably lysosomal proteases stimulated DR4 internalization via activation of caspases and their contribution became insignificant when DR5-dependent caspase activation reached maximum. There is growing evidence that non-caspase proteases, in particular lysosomal cathepsins, can play an important role in the regulation of apoptosis [31]. It was shown that in human pancreatic carcinoma cells bortezomib disrupted lysosomes with release of cathepsin B to the cytosol where it cleaved caspase-2 and induced mitochondrial apoptotic pathway [32]. Redistribution of the cathepsin B from lysosomes to the cytosol in cholangiocarcinoma cells was induced by TRAIL and contributed significantly to apoptosis [33], [34]. In our model E-64 and Baf-A1 did not significantly affected cell death induced by TRAIL but strongly inhibited the sensitizing effect of bortezomib for either TRAIL or DR5-B variant mediated cell death (Fig. 7). Probably activation of cathepsins amplified DR5-dependent apoptotic pathway.

It is paradoxical but our results indicated that the depletion of DR4 receptor was necessary for DR5 activation. This hypothesis is supported by the observed time-dependent increase in efficiency of DR5-B variant induced by bortezomib in HCT116 p53^−/−^ cells (Fig. 2B). During 16 h of co-treatment with TRAIL and bortezomib the level of DR4 at the cell surface remained high and sensitizing effect of bortezomib was negligible for DR5-B in comparison to wild type TRAIL. However, after 16 h when DR4 receptor was practically vanished from the cell surface pro-apoptotic efficiency of DR5-B became 10 fold higher than of wild type TRAIL. In agreement with our hypothesis it was shown recently that silencing of DR4 in HCT116 p53^+/+^ cells significantly stimulated apoptosis induced by TRAIL in combination with 5*-*Fluorouracil [35]. The same phenotypic effect was observed in MDA-MB-231 cells (Fig. S2, S3). It is not clear why evolution has created two death receptor with the same function. Although it was demonstrated that combined treatment of cancer cells with monoclonal antibodies to both death receptors only slightly enhanced the efficiency of cell death comparing when they were used separately [36]. Even our data are not strong evidence but we would hypothesize that DR4 receptor can play some regulatory role in DR5 mediated apoptosis.

In conclusion, it should be mentioned that when combination of bortezomib and TRAIL strongly stimulated HCT116 cell death the surviving cells became even more resistant to TRAIL since the ratio of the death and the decoy receptors dramatically decreased independently of p53 status (Fig. 3C). These observations support the therapeutic strategy to choose higher doses of TRAIL to kill as much cells as possible and to escape the residual resistance (Fig. 2B). Our results indicated that before application of the death receptor specific agents (either monoclonal antibodies or receptor specific mutant variants of TRAIL) in combination with chemotherapeutic drugs the receptor selectivity of the cells should be checked carefully. It is clear from our study that modification of the receptors expression induced by combined treatment can strongly differ from that induced by the same agents separately. It is important to investigate the molecular determinants of this phenomenon to choose optimal death receptor targeting agents and sensitizers in combined treatment of cancer.

## Supporting Information

Figure S1 **Trypsin does not cleave TRAIL receptors at the cell surface.** HCT116 p53^+/+^ cells were detached by trypsin-EDTA solution, 0.6 mM EDTA or by citric saline buffer and the level of constitutive surface expression of the death and decoy receptors were determined by flow cytometry. TRAIL receptors cell surface expression was analyzed using FITC-conjugated mouse anti-TRAIL-R1 (DR4), anti-TRAIL-R2 (DR5), anti-TRAIL-R3 (DcR1) and anti-TRAIL-R4 (DcR2) antibodies (Abnova). Mouse IgG (Immunotech) was used as isotype control. Cells (at least 1×10^5^ cells for each sample) were incubated with 1 µg of antibodies for 1 h at 4°C, washed in ice-cold PBS twice, resuspended in FACS buffer containing 0.5 µg/ml propidium iodide and analyzed by FACScan flow cytometer using Cellquest software (Becton Dickinson). Values in all experiments are mean ± SD of at least three independent experiments.
(TIF)

Figure S2 **Time-dependent expression of the death and the decoy receptors at the surface of MDA-MB-231 cells treated by bortezomib and TRAIL.** In all experiments, cells were treated with 25 nM bortezomib and 1 ng/ml TRAIL alone or in combination at indicated periods and the level of death and decoy receptors cell surface expression was analyzed using FITC-conjugated mouse anti-TRAIL-R1 (DR4), anti-TRAIL-R2 (DR5), anti-TRAIL-R3 (DcR1) and anti-TRAIL-R4 (DcR2) antibodies (Abnova) by FACScan flow cytometer using Cellquest software (Becton Dickinson). Values in all experiments are mean ± SD of at least three independent experiments.
(TIF)

Figure S3 **Time-depended influence of bortezomib on TRAIL or DR5-B mediated cell death in MDA-MB-231 cells.** (A) Contribution of death receptors DR4 and DR5 in TRAIL-mediated cell death in MDA-MB-231 cells. Cells were pre-incubated with 20 µg/ml antagonistic antibodies to death receptors or IgG1 control for 1 h following 4 h treatment with TRAIL or DR5-B (500 ng/ml) and cell death was determined by MTT test. Values are mean ± SD of at least three independent experiments. (B) Viability of the cells treated with different concentrations of TRAIL or DR5-B with or without bortezomib (25 nM) during 8, 16, 20 and 24 h of incubation. Values are mean ± SD of at least three independent experiments.
(TIF)
