# Supplementary figures and images for: Combination of TRAIL with Bortezomib Shifted Apoptotic Signaling from DR4 to DR5 Death Receptor by Selective Internalization and Degradation of DR4
Source: PLoS One. 2014 Oct 13;9(10):e109756. doi: 10.1371/journal.pone.0109756 (PMC4195680; doi:10.1371/journal.pone.0109756)

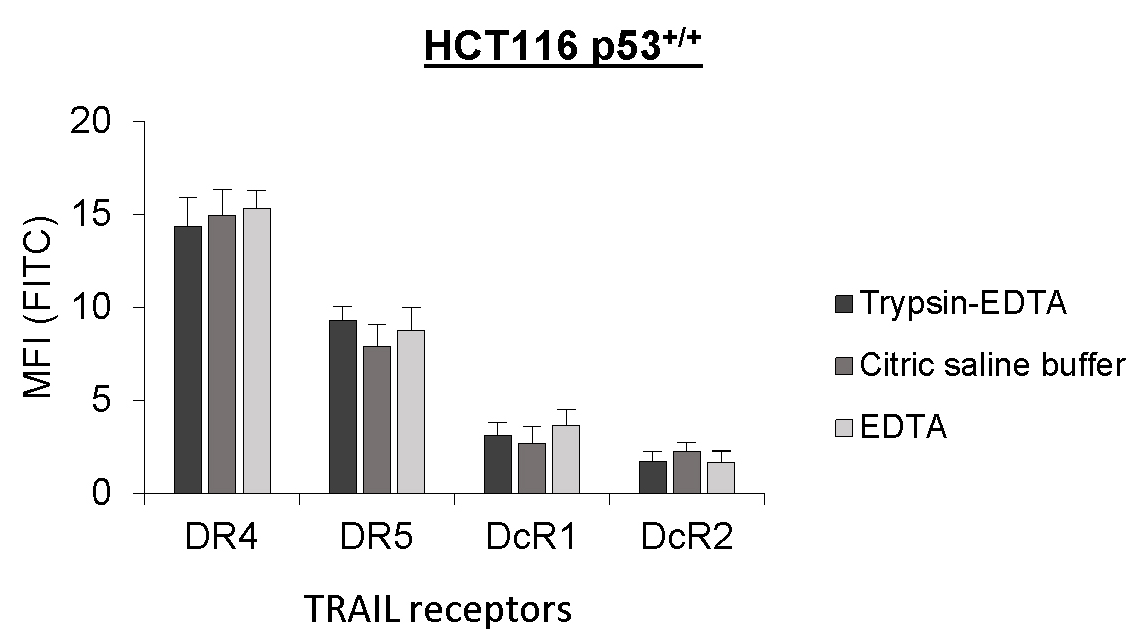

Supplement: Figure S1 — Trypsin does not cleave TRAIL receptors at the cell surface. HCT116 p53+/+ cells were detached by trypsin-EDTA solution, 0.6 mM EDTA or by citric saline buffer and the level of constitutive surface expression of the death and decoy receptors were determined by flow cytometry. TRAIL receptors cell surface expression was analyzed using FITC-conjugated mouse anti-TRAIL-R1 (DR4), anti-TRAIL-R2 (DR5), anti-TRAIL-R3 (DcR1) and anti-TRAIL-R4 (DcR2) antibodies (Abnova). Mouse IgG (Immunotech) was used as isotype control. Cells (at least 1×10^5^ cells for each sample) were incubated with 1 µg of antibodies for 1 h at 4°C, washed in ice-cold PBS twice, resuspended in FACS buffer containing 0.5 µg/ml propidium iodide and analyzed by FACScan flow cytometer using Cellquest software (Becton Dickinson). Values in all experiments are mean ± SD of at least three independent experiments. (TIF) [file pone.0109756.s001.tif]

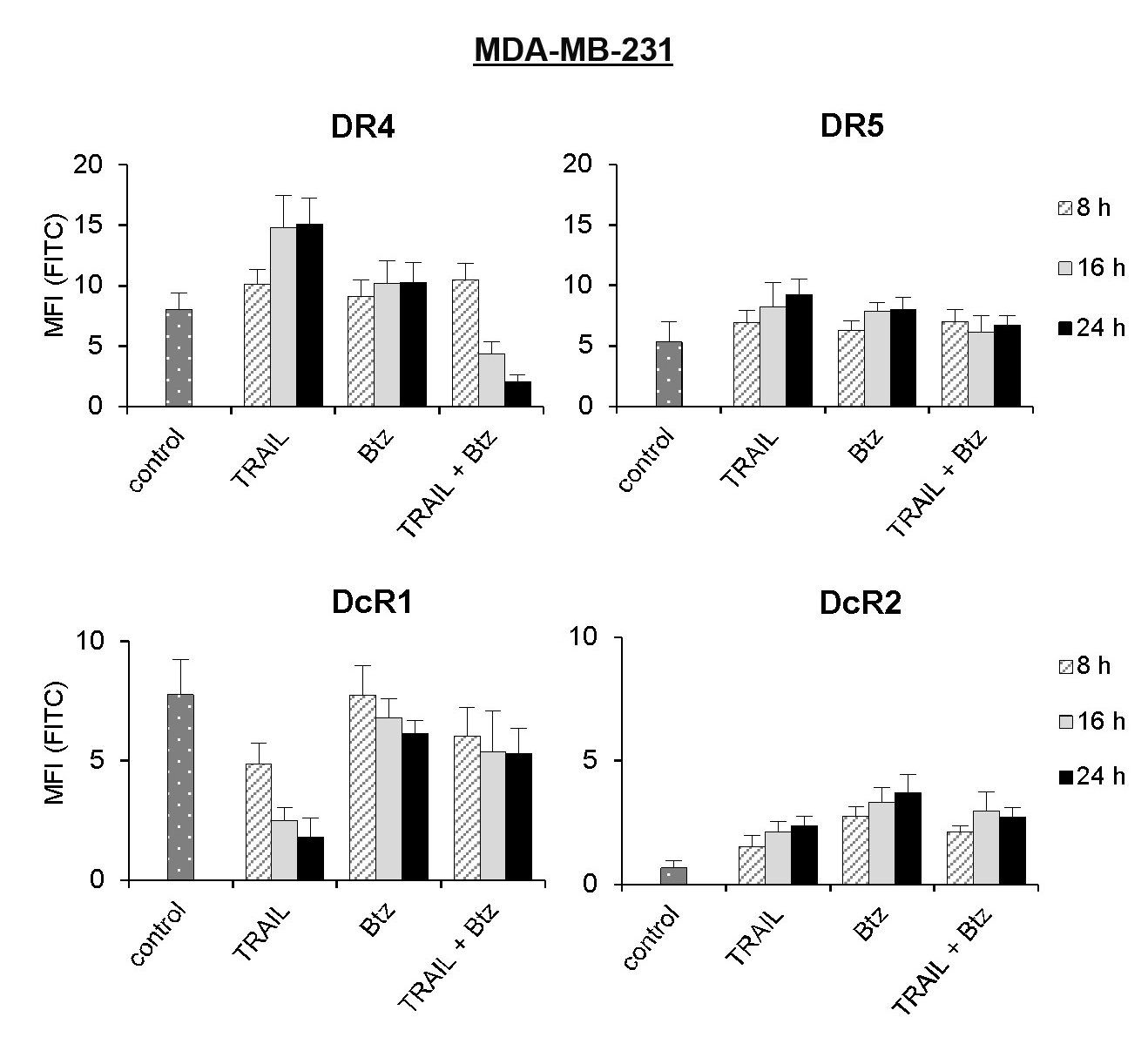

Supplement: Figure S2 — Time-dependent expression of the death and the decoy receptors at the surface of MDA-MB-231 cells treated by bortezomib and TRAIL. In all experiments, cells were treated with 25 nM bortezomib and 1 ng/ml TRAIL alone or in combination at indicated periods and the level of death and decoy receptors cell surface expression was analyzed using FITC-conjugated mouse anti-TRAIL-R1 (DR4), anti-TRAIL-R2 (DR5), anti-TRAIL-R3 (DcR1) and anti-TRAIL-R4 (DcR2) antibodies (Abnova) by FACScan flow cytometer using Cellquest software (Becton Dickinson). Values in all experiments are mean ± SD of at least three independent experiments. (TIF) [file pone.0109756.s002.tif]

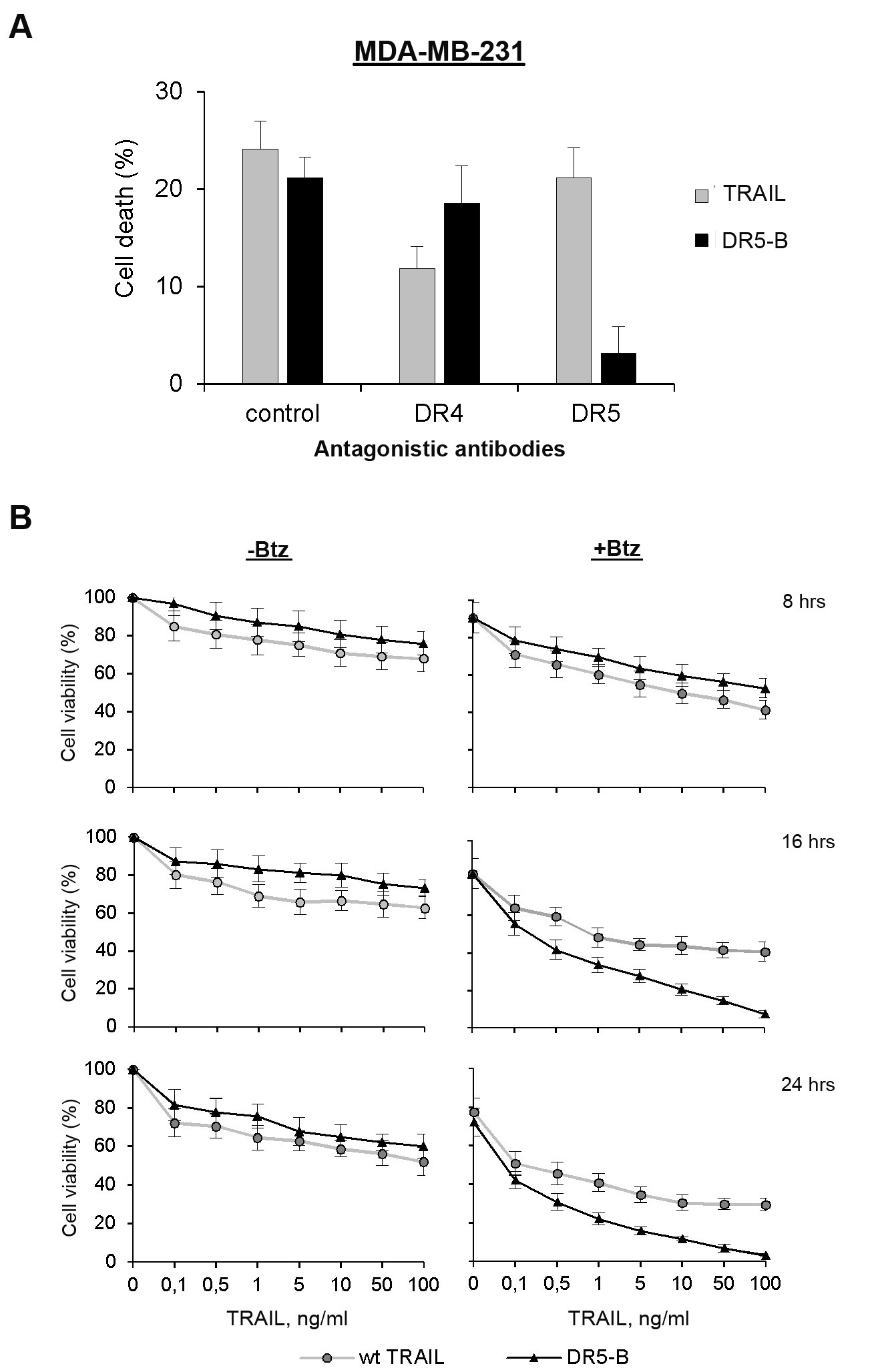

Supplement: Figure S3 — Time-depended influence of bortezomib on TRAIL or DR5-B mediated cell death in MDA-MB-231 cells. (A) Contribution of death receptors DR4 and DR5 in TRAIL-mediated cell death in MDA-MB-231 cells. Cells were pre-incubated with 20 µg/ml antagonistic antibodies to death receptors or IgG1 control for 1 h following 4 h treatment with TRAIL or DR5-B (500 ng/ml) and cell death was determined by MTT test. Values are mean ± SD of at least three independent experiments. (B) Viability of the cells treated with different concentrations of TRAIL or DR5-B with or without bortezomib (25 nM) during 8, 16, 20 and 24 h of incubation. Values are mean ± SD of at least three independent experiments. (TIF) [file pone.0109756.s003.tif]
